# Brazilian national telediagnosis platform: a data report for scientific research and public health improvement

**DOI:** 10.3389/fdgth.2026.1766606

**Published:** 2026-02-05

**Authors:** Walter Soares B. R. Filho, Letícia A. N. de Sousa, Heider A. Pinto, Raphael S. Fontes, Gleyson J. P. Caldeira Silva, Felipe Fernandes, Ingridy Barbalho, Antonio H. F. de Morais, Ernano Arrais Júnior, Tiago de Oliveira Barreto, Cesar A. Teixeira, Jorge Henriques, Guilherme Medeiros Machado, Janaína Valentim, Karilany D. Coutinho, Ricardo Valentim

**Affiliations:** 1 Laboratory for Technological Innovation in Health (LAIS), Federal University of Rio Grande do Norte (UFRN), Natal, Rio Grande do Norte, Brazil; 2 Graduate Program on Electrical and Computer Engineering, Federal University of Rio Grande do Norte, Natal, Rio Grande do Norte, Brazil; 3 Advanced Nucleus for Technological Innovation (NAVI), Federal Institute of Rio Grande do Norte (IFRN), Natal, Rio Grande do Norte, Brazil; 4 University of Coimbra, Health Sciences Research Unit: Nursing (UICISA: E), Nursing School of Coimbra (ESEnfC), Coimbra, Portugal; 5 Department of Informatics Engineering, University of Coimbra, Center for Informatics and Systems of the University of Coimbra, Coimbra, Portugal; 6 LyRIDS Lab, Ecole Centrale d’Electronique (ECE), Paris, France; 7 Centre for Global Studies (CEG), Open University, Lisboa, Portugal

**Keywords:** cardiovascular diseases, electrocardiogram (ECG), epidemiological surveillance, public health, telehealth

## 1 Introduction

Cardiovascular diseases are the leading causes of death globally [1], with myocardial ischemia and cerebrovascular accident (CVA) the most prevalent, accounting for 13% and 10% of total deaths worldwide, respectively [2]. Since the 2000s, a progressive increase in these diseases has been observed, which are adding to global concern as they are chronic noncommunicable diseases (NCDs) with a high health and social burden. This scenario has driven the formulation of public policies and the development of technological solutions to reduce morbidity and mortality associated with cardiovascular disease [3, 4].

Although cardiovascular diseases are a global health issue [5], each country has its own peculiarities and challenges, which requires epidemiological studies capable of identifying specific causes and supporting more effective public health interventions [6]. In the Brazilian scenario, such challenges are compounded by the country’s vast territory, large population, and remarkable sociocultural and economic diversity. Brazil has an area of 8,516,000 ${\mathrm{km}}^{2}$, distributed across 26 states, one Federal District, and 5,569 municipalities, and has population of 212 million inhabitants [7]. Altogether, these factors are decisive in determining the population’s eating patterns and lifestyle [6, 8].

These factors, coupled with regional inequalities and cultural syncretism, have direct influence on dietary habits, lifestyle, and, consequently, the population’s health status. Given this complexity, policymakers and public managers are faced with challenges that extend beyond the simple implementation, execution, and coordination of programs and initiatives focused on cardiovascular health promotion and care [9].

In this vein, it is essential to continuously invest in health education initiatives and public health interventions that strengthen epidemiological surveillance, improve population health monitoring, and enable faster, more effective, and more efficient clinical responses. Such measures are key in reducing mortality from cardiovascular diseases in the country, many of which are preventable [10].

Furthermore, it is worth noting that cardiovascular diseases, even when not fatal, often result in disability and long-term sequelae, imposing a high financial and social burden on health systems [11–13]. In several countries, digital health has been consolidated as a strategic field with the capacity to mitigate the effects of cardiovascular diseases, particularly in the field of diagnosis, electronic patient records, and decision support [14–17]. These dimensions of digital health provide a deeper understanding of individuals’ clinical characteristics, behaviors, and lifestyles, which is important for public health authorities to better comprehend the epidemiological profile of the population at local, regional, and national levels.

In this scenario, the implementation of public health policies that promote research, technological development, and innovation has become increasingly essential. Such strategies have the potential to reduce mortality, promote greater resilience and responsiveness in countries’ health systems, and contribute to the sustainability and efficiency of prevention and care measures [14, 18].

Considering Brazil’s reality and the context of cardiovascular diseases, telediagnosis has produced positive results. This is notably the case for populations living far from major urban centers and with limited access to specialized services, enabling more timely diagnoses, interventions, and screenings [19, 20].

Among South American countries, Brazil stands out as a country with continent-sized dimensions and significant regional, sociocultural, and economic disparities [21]. It features one of the largest public health systems worldwide, known as SUS, with universal coverage available free of charge to the entire population [22].

In countries like Brazil, with a vast territory, large population, and marked income inequality, especially in certain regions [23], it is to be expected that health services will face challenges in providing equitable care across the entire population. In this context, it became necessary to design, develop, implement, and integrate a digital health solution capable of mitigating geographic barriers and asymmetries while promoting equitable access to health services, particularly specialized healthcare. The National Telediagnosis Platform (PNTD) [20, 24–26] was created to address these challenges and is available at: https://pntd.telessaude.ufrn.br/ptd.

This platform was developed within the Telessaúde Brasil Program to meet the demands of Brazil’s National Health System (SUS), with the primary aim of expanding and promoting equitable access to health services nationwide [27–29]. As a digital health solution, PNTD is relevant not only for the healthcare field but also serves as an invaluable source of data for public health managers, policymakers, epidemiologists, and researchers [28, 30, 31].

The PNTD has been operating in Brazil since 2017 and covers more than 1,300 municipalities in eleven Brazilian states. According to data available on the PNTD website, more than 1,746,912 electrocardiograms (ECGs) have been performed between its creation and the end of 2023. The platform also compiles information that reflects the Brazilian diversity, gathering data on behavior, eating habits, ethnicity, morphology, and remote biomedical testing, thereby constituting a unique basis for innovative public health research.

In technical terms, the PNTD uses a microservices architecture built with open-source software (PHP, Laravel, PostgreSQL), which ensures flexibility, security, and scalability. Its RESTful API facilitates integration and interoperability between across healthcare systems. Security is ensured via HTTPS encryption, and the use of JSON for data structuring streamlines communication between systems [28].

The national provision of telediagnosis services seeks to expand access to diagnostic tests that can be performed remotely, particularly in priority areas of the country. This initiative is coordinated by the Brazilian National Telehealth Program, under Brazil’s Ministry of Health, and involves collaboration between State and Municipal Health Departments, State and Specialized Telehealth Centers, and Telediagnosis Points located within healthcare facilities. This coordinated effort aims to optimize the coverage and efficiency of diagnostic care for populations in remote and underserved areas [20, 32].

In light of the above, this data report aims to present and make available the ECG dataset acquired through the National Telediagnosis Platform, describing its collection, organization, and formatting for use. This large volume of clinical and demographic information represents a robust sample of the Brazilian population, enabling analyses of critical variables such as gender distribution, age groups, geographic dispersion, and comorbidities, while also contributing to the formulation of evidence-based public policies. Moreover, this data report aims to deepen the understanding of population health patterns and support the development of innovative public health strategies.

## 2 Methods and materials

This data report structured data from 1,746,912 clinical ECG tests, evaluated and labeled by cardiologists, between September 2017 and December 2023. The tests were carried out in more than 1,300 Brazilian municipalities, covering different regions of the country and reflecting the wide reach of the national telediagnosis service. To properly understand the nature, composition, and structure of the data used in this data report, it is essential to understand how the National Telediagnosis Platform operates. The following is a brief description of the data acquisition and processing flow by PNTD:

The patient is admitted to a healthcare facility (telediagnosis center) in one of the locations in Brazil equipped for this purpose.
Following this, an electrocardiogram technician performs the cardiac test and obtains the cardiac data record together with the patient’s information.
The test is performed and sent to PNTD.
The PNTD regulates the patient’s test by referring them to a cardiologist who will receive and perform the report remotely. Upon receiving the test data, the platform identifies, among the registered cardiologists, one who is immediately available to perform the electrocardiogram report. The resulting report is then returned to the requesting healthcare facility, which, depending on the results, proceeds with the appropriate clinical actions.

At the end of the flow described, the data are stored in a Postgres database so that it can be accessed for different purposes, whether for data consultation or research. The dataset used in this study was extracted from the National Telediagnosis Platform database and underwent an anonymization process to ensure confidentiality and privacy, in accordance with Brazil’s General Data Protection Law (LGPD) [33].

This anonymized database was subjected to ETL (Extract, Transform, Load) techniques that support data discovery, report generation, analysis, and decision making [34]. In activities involving large volumes of data, where it is necessary to ensure the reliability of samples, ETL steps prove to be an essential and necessary stage [35]. At the end of the ETL process, the data are expected to be ready for use for its intended purpose, without missing data or inconclusive information that could compromise the analysis or final results of the research.

Importantly, in addition to their use in databases with large volumes of data, ETL processes contribute significantly to the improvement of business intelligence practices and data analysis, yielding comparable effects when applied to big data analysis and machine learning, i.e., they make the process more reliable, accurate, detailed, and efficient [36]. In this sense, for business intelligence purposes, the research used Microsoft Power BI, a software widely used by the academic and corporate communities for data analysis, pattern identification, and accurate and reproducible information extraction [37].

The first stages of data structuring aimed to remove information that clearly contained errors or flaws, such as incorrect birth date records with past or future years out of context—in the same sense as the date of examinations. Incomplete records or entries incorrectly registered as tests were also removed.

Other adjustments included data type conversions and structural reorganizations to improve the database structure. For this purpose, Power BI’s native ETL tool, Power Query, was used. This tool uses the M language for data modeling and processing.

**Figure 1:**
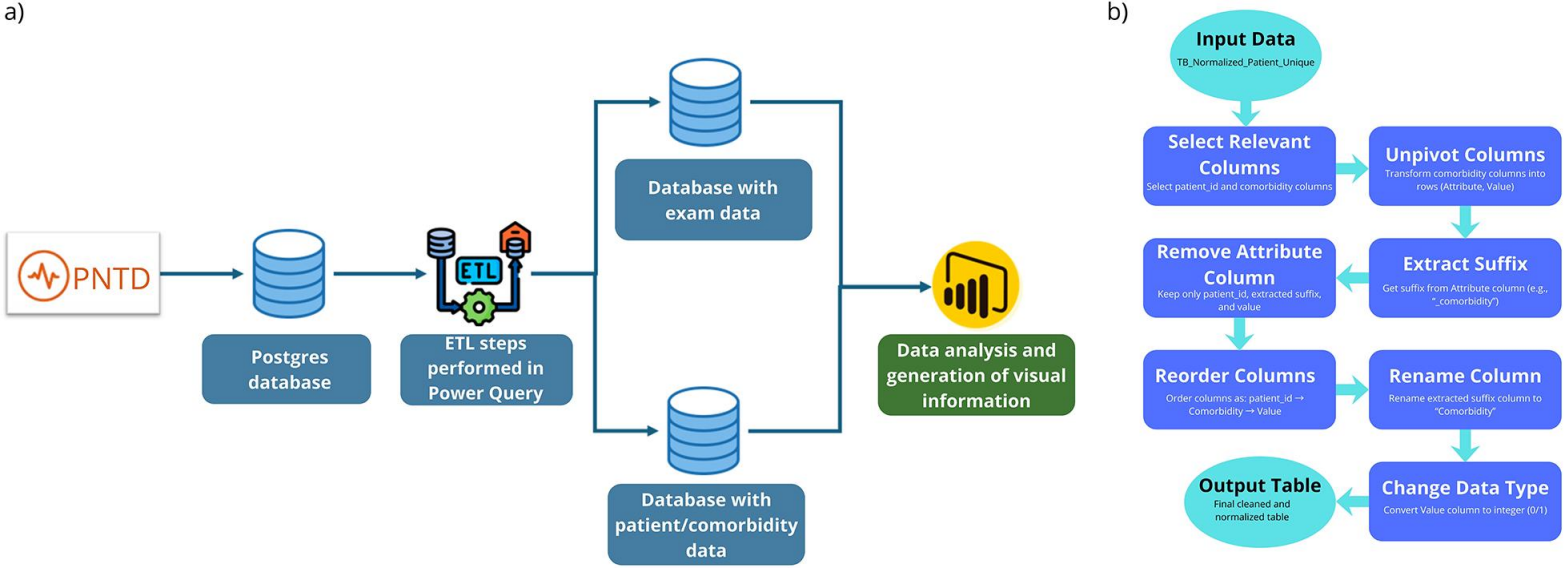
**(a)** Data processing workflow. **(b)** Detailed data processing flow. Description of processing algorithm. Icons from: “Free Database icon” by phatplus, licensed under Flaticon License; “Free Etl icon” by Freepik, licensed under Flaticon License; “Power BI Business intelligence Microsoft Corporation Data visualization Data analysis” by HiClipart (https://www.hiclipart.com/terms-of-use).

The Supplementary Material (Algorithm 1.1) and Figures 1a, b used during data processing to generate the examination data table. To create the patient table, i.e., a table that contains only the most recent test for each patient and is used to study comorbidities, the following excerpt was added to the aforementioned Algorithm 1.2 in the Supplementary Material.

In the case of comorbidities, the information was distributed across different fields, making aggregate analysis difficult. Therefore, these data were integrated into a single column called comorbidities, allowing for a more consistent visualization. In addition, the data were aggregated into numeric (quantitative) variables, which facilitated the reading and interpretation of the results, with the script being as represented in Algorithm 1.3. in the Supplementary Material.

After these steps, it was possible to transform raw data into useful information for the research, ensuring that the analytical process is supported by reliable and consistent data. Each stage of ETL (see Figure 1a), from anonymization and cleaning (data sanitization) to transformations and aggregations, contributed to the result of a solid foundation, enabling accurate insights into patient profiles. Thus, the methodological approach adopted not only facilitated data exploration, but also provided a secure foundation for future analyses and more informed decision-making.

Next, Figure 1b illustrates the workflow for a clearer understanding of the process, from data acquisition from PNTD, through all ETL processing, and finally, obtaining information and presenting it in the Power BI Business Intelligence tool.

The data processing flow begins with the import of a normalized patient table containing a single record per individual. From this dataset, only the relevant columns were selected, specifically the patient identifier and fields related to comorbidities. Next, these columns of comorbidities are transformed from a wide format to a long format using the unpivot operation, which converts columns into rows, reorganizing the table to leave the data in a long format, thus resulting in two columns, one representing the name of the comorbidity (“Attribute”) and the other containing the corresponding value (e.g., 0, 1, or null). Next, the suffix of each comorbidity name is extracted to isolate the specific name of the condition, simplifying the attribute into a cleaner and more intuitive label.

After extracting the suffix, the redundant “Attribute” column is removed and the table columns are reorganized to follow a standardized structure: patient identifier, comorbidity name, and value. The suffix column is then renamed to “Comorbidity” for clarity, and the “Value” column is converted to the integer data type, ensuring consistency in subsequent analyses. The result is a clean, well-structured dataset, where each row represents a unique patient–comorbidity pair, ready for statistical or clinical analyses.

## 3 Descriptive analysis

The database analyzed comprises 1,746,912 (one million, seven hundred forty-six thousand, nine hundred twelve) ECG tests performed on 1,422,587 (one million, four hundred twenty-two thousand, five hundred eighty-seven) unique patients (single identifier).

This study is grounded in a quantitative analysis of variables related to ECG tests and the demographic and clinical characteristics of the patients assessed. The information analyzed was obtained from spontaneous medical history interviews conducted at the time of the test.

**Figure 2:**
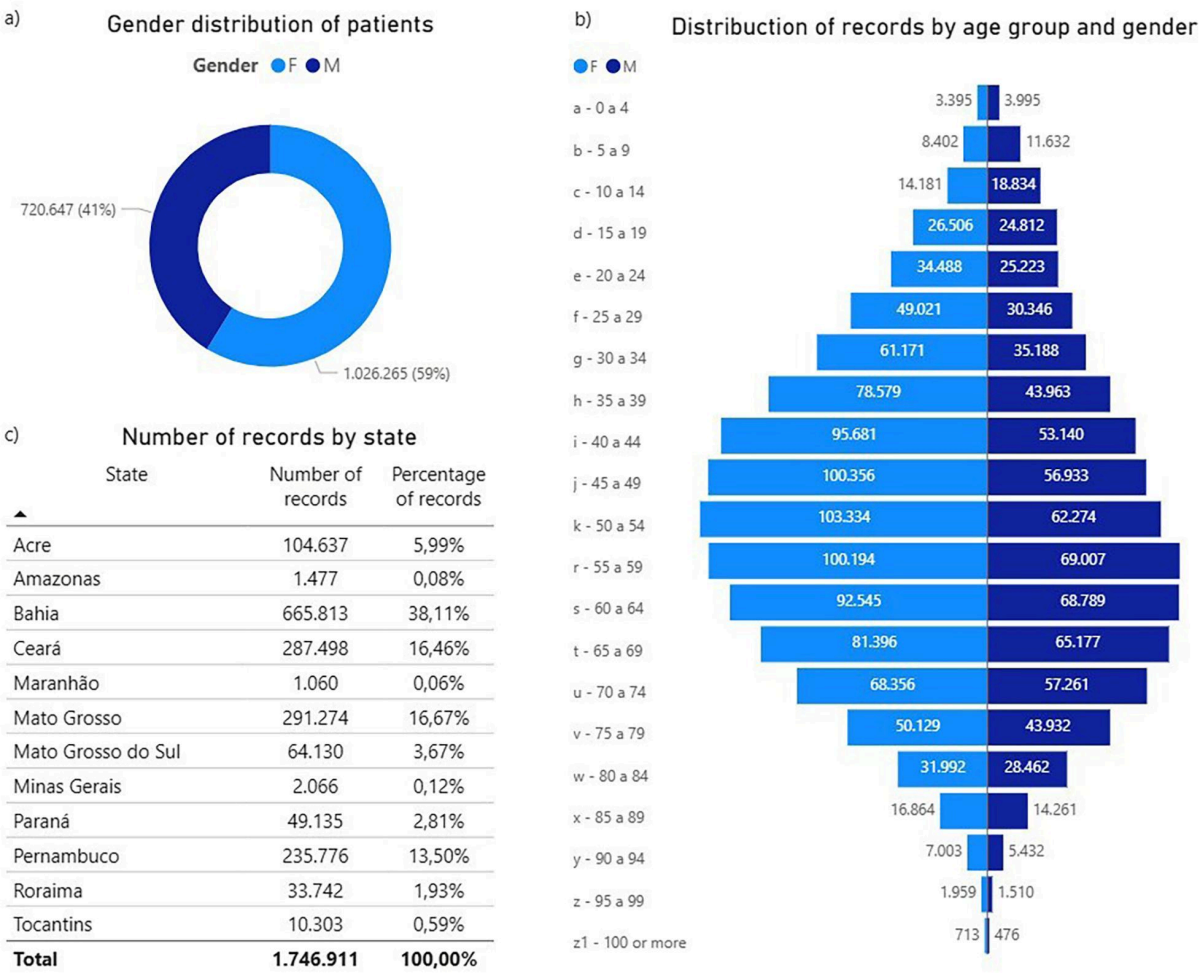
**(a)** Patient gender distribution; **(b)** Age-group and gender distribution of records; **(c)** Number of records by state (Federative Unit).

The tests analyzed were performed between 2017 and 2023, spanning multiple states in Brazil. Records were distributed by state as follows: Acre (104,637 tests), Amazonas (1,477 tests), Bahia (665,813 tests), Ceará (287,498 tests), Maranhão (1,060 tests), Mato Grosso (291,274 tests), Mato Grosso do Sul (64,130 tests), Minas Gerais (2,066 tests), Paraná (49,135 tests), Pernambuco (235,776 tests), Roraima (33,742 tests), and Tocantins (10,303 tests). Figure 2c shows quantitative records by Brazilian states.

In this context, the demographic profile related to gender distribution were examined. The proportion of males and female patients was observed, along the distribution per age group, in order to understand the composition and diversity of the population (Figure 2b). As a result, the number of tests performed on female patients is markedly higher than that performed on males. Notably, the data suggest that male patients tend to seek healthcare services, mostly, later in life, around the age range of 55 to 64 years old. Meanwhile, females generally seek health care from approximately 40 years onward—i.e., at least 15 years earlier than males.

Another noteworthy finding regarding gender and age is that, in the 45–49 age group, females account for nearly twice as many tests as males. This indicates the need for educational and health-promotion campaigns aimed at encouraging men to seek medical care earlier in life.

From this perspective, it can be noted that female patients accounted for the majority of ECG tests, comprising 59% of consultations, while male individuals accounted for 41%, as shown in Figure 2a.

Further relevant information can be extracted from analyzed data and refers to past comorbidities and/or medical history reported by patients. As shown in Figure 2a, arterial hypertension is the predominant comorbidity among patients, affecting 594,381 patients, which represents a 41% prevalence. This comorbidity precedes diabetes mellitus, with 185,069 patients (13%), and a family history of coronary heart disease, present in 169,821 patients (11%). These figures are noteworthy, given their consistency with international and national studies [31].

Patients have only one comorbidity (almost 60% of the group with ⩾1, approximately 469,000 patients), a significant number of patients have two comorbidities (approximately 28%), and the groups with three, four, and over four comorbidities form the tail of the distribution (≈9%, ≈3%, and ≈1%, respectively). In all categories, the number of males exceeds that of females. For instance, among those with a single comorbidity, there are 281,800 males compared to 186,800 females, suggesting a higher concentration of factors in male patients. Considering only those who have at least one condition, the implied average–simple average calculation automatically generated by Power BI—is around 1.8 comorbidities per patient, thereby confirming a profile predominantly of one to two comorbidities per individual.

In terms of geographic distribution, there is considerable heterogeneity between the states: some concentrate very high volumes, while others show residual numbers. This is more indicative of differences in coverage/registration in the database rather than necessarily real differences in prevalence or incidence. Therefore, direct comparisons between Brazilian states should be made with caution and, ideally, normalized by the number of patients in each state (e.g., rate per thousand patients).

Nevertheless, the relative pattern within states that hypertension tends to be more prevalent, followed by diabetes, family history of coronary artery disease (CAD), smoking, and obesity, while conditions such as previous cerebrovascular accident (CVA)/heart attack, chronic obstructive pulmonary disease (COPD), and chronic kidney disease (CKD) occur less frequently.

In light of the above, it can be concluded that the analysis of 1,746,912 ECG tests performed on 1,422,587 unique patients in more than 1,300 Brazilian municipalities confirms that the National Telediagnosis Platform fulfills a dual strategic role: it democratizes access to cardiac care in Brazil’s National Health System and, simultaneously, enables epidemiological surveillance on a national scale. The profile observed is typical of cardiometabolic risk, with a predominance of high blood pressure, diabetes mellitus, family history of coronary heart disease, smoking, and obesity. Most individuals have one to two comorbidities, and males have a higher number of factors in all age groups, in addition to their tendency to seek health care later than females. In part, the differences between states reflect asymmetries in coverage and registration, highlighting the need for the use of standardized indicators (e.g., per thousand patients) for comparisons and territorial planning.

From a methodological standpoint, the anonymization process in compliance with Brazil’s General Personal Data Protection Law, combined with the ETL (Extract, Load, Transform) stages and the use of Power BI, ensured the quality and reproducibility of the experiment and the evidence. Therefore, it allowed routine healthcare data to be transformed into actionable information for public health management. These results highlight clear public policy priorities: strengthening initiatives for the prevention and control of hypertension, diabetes, smoking cessation, and obesity management; ramping up specific campaigns for younger men; and improving the standardization of records to reduce information bias.

In summary, Brazil’s PNTD is a scalable, secure, and interoperable infrastructure capable of increasing equity in access to diagnosis, informing timely clinical decisions, and supporting evidence-based public health policies. Furthermore, it produces valuable data for academic studies, whose potential for research development represents a major contribution to the scientific community. This is especially noteworthy at this time, when qualified data are needed for training artificial intelligence-based systems, which are applied in a multitude of healthcare settings.

This data report presents a selection of relevant data on cardiovascular health within Brazil, demonstrating the profile of patients and their associated comorbidities. The legitimacy of this information is reinforced by its collection through the telediagnosis platform, a structured data source directly linked to Brazilian public health services. In the specific case of this manuscript, the sample covers more than 1,300 municipalities throughout Brazil. The availability of the database in an open and structured format is a differential that greatly facilitates its reuse by researchers, managers, and health professionals, thus promoting transparency and scientific collaboration on a global scale. Consequently, the database can spur new research and analysis to support the formulation of more effective public policies and strategies for cardiovascular health care in Brazil. Finally, the quality and usefulness of the information generated highlight the importance of developing and implementing solutions such as the National Telediagnosis Platform, which enables more efficient and reliable data collection on a national scale, a strategic digital health solution for epidemiological surveillance and the continuous improvement of public health.

In the context of artificial intelligence and the application of advanced computational methods, this data report is of significant value, as it presents a rich database validated by a group of healthcare experts and properly processed by healthcare data scientists. Therefore, it can be used for a multitude of research and studies. For instance, it can be used to develop predictive models applied to the development of epidemiological scenarios, as well as for individual patient health. It is an original database that can be used in a wide range of research and studies.

## Data Availability

The datasets presented in this study can be found in online repositories. The names of the repository/repositories and accession number(s) can be found below: https://zenodo.org/records/17899762?preview=1&amp;token=eyJhbGciOiJIUzUxMiJ9.eyJpZCI6ImQwYmE4MGRhLTgwYzctNDdjMS1hNDQ4LTVhNDEwMDgzY2QxZSIsImRhdGEiOnt9LCJyYW5kb20iOiJjMmYzZGYwZTIwNGM5MTNmMWRhN2UyNmZkOTUwOGExYiJ9.y1A_sFs67RiGHF9MhdysCwRy-y2rfk5KbzpWXUdOORj0IFXnM-00H7SkhhUTaJR27sxW43oKLRmCR6J-C2VJUQ.
