# Supplementary material for: Brazilian national telediagnosis platform: a data report for scientific research and public health improvement
Source: Front Digit Health. 2026 Feb 5;8:1766606. doi: 10.3389/fdgth.2026.1766606 (PMC12917772; doi:10.3389/fdgth.2026.1766606)
Supplement: Supplementary file 1 [file Datasheet1.pdf]

frontiers

Supplementary Material

1 ALGORITM

1.1 Algorithm generated during data processing that produced the examination table

```
let
    // === Data source: the table already loaded/defined in the model ===
    SourceReports = TB_Laudos,

    // === Removal of irrelevant columns for the analysis ===
    IrrelevantColumnsRemoved =
        // Remove from the dataset fields that will not be used later
        Table.RemoveColumns(
            SourceReports,                                    // input table
            {
                "cnesNucleo", "dataRecepcao", "entregaParaLaudagem",
                "fila_exame_descricao", "fila_exame_prioridade", "fila_exame_ufreferencia",
                "tipo_exame_id", "historico_paciente_idsync", "historico_paciente_nascimento",
                "historico_comorbidade_id", "historico_medicamento_id", "historico_medicamento_idsync",
                "responsavel_cbo", "responsavel_sexo", "responsavel_ativo", "responsavel_docuf",
                "responsavel_funcao_id", "responsavel_funcao_descricao", "responsavel_docemissor",
                "solicitante_sexo", "solicitante_docuf", "solicitante_funcao_id",
                "solicitante_funcao_descricao", "solicitante_docemissor",
                "status_exame_descricao", "status_exame_tipostatus",
                "especialidade_descricao", "estabelecimento_cns",
                "estabelecimento_municipio_uf_ibge", "prioridade_exame_descricao",
                "laudo_exame_ativo", "laudo_idespecialista"
            }                                                  // list of columns to remove
        ),

    // === Filter by exam date (2017-01-01 to 2023-12-31) ===
    ExamPeriodFiltered =
        Table.SelectRows(
            IrrelevantColumnsRemoved,                         // input table
            each [dataRealizacao] >= #datetime(2017,1,1,0,0,0)
             and [dataRealizacao] <= #datetime(2023,12,31,0,0,0) // filter condition
        ),

    // === Adjust data type of patient's birth date to Date ===
    TypesAdjusted =
        Table.TransformColumnTypes(
            ExamPeriodFiltered,                               // input table
            {{"paciente_nascimento", type date}}              // type conversion
        ),

    // === Remove known invalid record (id 647081) ===
    InvalidRecordRemoved =
        // Note: this id has patient_birth in an incorrect format
        Table.SelectRows(
            TypesAdjusted,                                    // input table
            each ([id] <> 647081)                             // keep all except the problematic id
```

```
        ),

    // === Calculate patients age on the exam date ===
    PatientAgeAdded =
        Table.AddColumn(
            InvalidRecordRemoved,                            // input table
            "patient_age",                                   // new column name
            each Duration.Days([dataRealizacao] - [paciente_nascimento]) / 365.25,
            type number                                      // resulting data type
        )
in

  PatientAgeAdded

  InvalidRecordRemoved,                                  // input table

    // === Add patient's age column ===
    PatientAgeAdded =
        Table.AddColumn(
            InvalidRecordRemoved,                           // input table
            "patient_age",                                  // new column name
            each                                            // row logic:
                if [paciente_nascimento] = null then -1     // if no birth date, mark as -1 (invalid)
                else
                    let
                        Today = [dataRealizacao],           // reference date (exam date)
                        Birth = [paciente_nascimento],      // birth date
                        Years = Date.Year(Today) - Date.Year(Birth) // simplified calculation by calendar year
                        // Note: if birthday precision is needed, adjust with month/day comparisons
                    in
                        Years,                              // returns the estimated age
            Int64.Type                                      // type of the new column
        ),

    // === Filter to keep only valid ages (0 to 125 years) ===
    ValidAgeFiltered =
        Table.SelectRows(
            PatientAgeAdded,                                // input table
            each [patient_age] >= 0 and [patient_age] <= 125
        ),

    // === Generate age group (letter + interval) according to rule ===
    AgeGroupAssigned =
        Table.AddColumn(
            ValidAgeFiltered,                               // input table
            "patient_agegroup",                             // new column
            each                                            // row logic:
                let age = [patient_age] in
                if age = null then null else
                if age <= 4  then "a - 0 to 4"   else
                if age <= 9  then "b - 5 to 9"   else
                if age <= 14 then "c - 10 to 14" else
                if age <= 19 then "d - 15 to 19" else
```

```
                if age <= 24 then "e - 20 to 24" else
                if age <= 29 then "f - 25 to 29" else
                if age <= 34 then "g - 30 to 34" else
                if age <= 39 then "h - 35 to 39" else
                if age <= 44 then "i - 40 to 44" else
                if age <= 49 then "j - 45 to 49" else
                if age <= 54 then "k - 50 to 54" else
                if age <= 59 then "r - 55 to 59" else
                if age <= 64 then "s - 60 to 64" else
                if age <= 69 then "t - 65 to 69" else
                if age <= 74 then "u - 70 to 74" else
                if age <= 79 then "v - 75 to 79" else
                if age <= 84 then "w - 80 to 84" else
                if age <= 89 then "x - 85 to 89" else
                if age <= 94 then "y - 90 to 94" else
                if age <= 99 then "z - 95 to 99" else
                "z1 - 100 or more",                         // label for 100+
            type text                                       // type of the new column
        ),

    // === Remove outlier patient with too many exams on the same day (patient_id = "144856") ===
    OutlierPatientRemoved =
        Table.SelectRows(
            AgeGroupAssigned,                               // input table
            each [paciente_id] <> "144856"                  // exclude this specific patient
        )

in
    // === Final query result ===
    OutlierPatientRemoved
```

1.2 Creation of the patient table. This table includes only the most recent test for each patient and is used for the analysis of comorbidities

```
// >>> Keep only the most recent record per patient_id <<<
#"SortedByPatientAndDate" = Table.Sort(#"FilteredRows3", {{"paciente_id", Order.Ascending}, {"
    dataRealizacao", Order.Descending}}),
#"LatestRecordPerPatient" = Table.Distinct(#"SortedByPatientAndDate", {"paciente_id"})
```

1.3 Integration of comorbidity data into a single column named comorbidities, enabling a more consistent visualization. The data were aggregated into numerical values

```
let
    // === Data source: normalized table with one row per patient ===
    UniquePatientsSource = TB_Normalized_Patient_Unique,

    // === Select only the columns of interest (id + comorbidity columns) ===
    ComorbidityColumnsSelected =
        Table.SelectColumns(
            UniquePatientsSource,                            // input table
            {                                                 // columns to keep
                "id",
                "historico_comorbidade_has",
```

```
                "historico_comorbidade_avcprevio",
                "historico_comorbidade_obesidade",
                "historico_comorbidade_tabagismo",
                "historico_comorbidade_displidemia",
                "historico_comorbidade_doencachagas",
                "historico_comorbidade_infartoprevio",
                "historico_comorbidade_usomarcapasso",
                "historico_comorbidade_diabetemelitus",
                "historico_comorbidade_angioplastiaprevia",
                "historico_comorbidade_doencarenalcronica",
                "historico_comorbidade_doencapulmonarcronica",
                "historico_comorbidade_revascularizacaomiocardiaprevio",
                "historico_comorbidade_historiafamiliardoencacoronariana"
            }
        ),

    // === Convert comorbidity columns into rows (unpivot) ===
    // Result: one column "Attribute" (comorbidity name) and another "Value" (0/1, null, etc.)
    ComorbiditiesToRows =
        Table.Unpivot(
            ComorbidityColumnsSelected,                         // input table
            {                                                   // columns to unpivot
                "historico_comorbidade_has",
                "historico_comorbidade_avcprevio",
                "historico_comorbidade_obesidade",
                "historico_comorbidade_tabagismo",
                "historico_comorbidade_displidemia",
                "historico_comorbidade_doencachagas",
                "historico_comorbidade_infartoprevio",
                "historico_comorbidade_usomarcapasso",
                "historico_comorbidade_diabetemelitus",
                "historico_comorbidade_angioplastiaprevia",
                "historico_comorbidade_doencarenalcronica",
                "historico_comorbidade_doencapulmonarcronica",
                "historico_comorbidade_revascularizacaomiocardiaprevio",
                "historico_comorbidade_historiafamiliardoencacoronariana"
            },
            "Attribute",                                        // column name for original headers
            "Value"                                             // column name for values
        ),

    // === Extract only the suffix after the second "_" from the comorbidity name ===
    // Example: "historico_comorbidade_has" -> "has" (occurrence = 1 starts after the 2nd "_")
    ComorbiditySuffixExtracted =
        Table.AddColumn(
            ComorbiditiesToRows,                                // input table
            "Text After Delimiter",                             // temporary new column
            each Text.AfterDelimiter([Attribute], "_", 1),       // extract text after 2nd "_"
            type text                                            // type of new column
        ),

    // === Remove the "Attribute" column (keep only the cleaned suffix) ===
    AttributeRemoved =
        Table.RemoveColumns(
```

```
            ComorbiditySuffixExtracted,                         // input table
            {"Attribute"}                                       // column to remove
        ),

    // === Reorder columns to id, comorbidity, and value (in that order) ===
    ColumnsReordered =
        Table.ReorderColumns(
            AttributeRemoved,                                   // input table
            {"id", "Text After Delimiter", "Value"}             // new order
        ),

    // === Rename the suffix column to "Comorbidity" ===
    ComorbidityNameStandardized =
        Table.RenameColumns(
            ColumnsReordered,                                   // input table
            {{"Text After Delimiter", "Comorbidity"}}           // rename mapping
        ),

    // === Change the "Value" column type to integer (0/1) ===
    TypesAdjusted =
        Table.TransformColumnTypes(
            ComorbidityNameStandardized,                        // input table
            {{"Value", Int64.Type}}                             // type conversion
        )

in
    // === Final result ===
    TypesAdjusted
```
